# Effects of FGF21‐secreting adipose‐derived stem cells in thioacetamide‐induced hepatic fibrosis

**DOI:** 10.1111/jcmm.13795

**Published:** 2018-07-18

**Authors:** Hwansu Kang, Eunhui Seo, Jong‐Moon Park, Na‐Young Han, Hookeun Lee, Hee‐Sook Jun

**Affiliations:** ^1^ College of Pharmacy and Gachon Institute of Pharmaceutical Science Gachon University Incheon Korea; ^2^ Lee Gil Ya Cancer and Diabetes Institute Gachon University Incheon Korea; ^3^ Gachon Medical Research Institute Gil Hospital Incheon Korea

**Keywords:** adipose‐derived stem cells, cell therapy, fibroblast growth factor 21, hepatic stellate cell, liver fibrosis

## Abstract

Mesenchymal stem cells (MSCs) have been investigated to treat liver diseases, but the efficiency of MSCs to treat chronic liver diseases is conflicting. FGF21 can reduce inflammation and fibrosis. We established FGF21‐secreting adipose derived stem cells (FGF21_ADSCs) to enhance the effects of ADSCs and transplanted them into thioacetamide (TAA)‐induced liver fibrosis mice via the tail vein. Transplantation of FGF21_ADSCs significantly improved liver fibrosis by decreasing serum hyaluronic acid and reducing the expression of fibrosis‐related factors such as α‐smooth muscle actin (α‐SMA), collagen and tissue inhibitor of metalloproteinase‐1 (TIMP‐1) compared with the Empty_ADSCs by inhibition of p‐JNK, NF‐κB and p‐Smad2/3 signalling. α‐lactoalbumin (LA) and lactotransferrin (LTF), secretory factors produced from FGF21_ADSCs inhibited TGF‐β1‐induced expression of α‐SMA and collagen in LX‐2 cells. These results suggest that transplantation of FGF21_ADSCs inhibited liver fibrosis more effectively than Empty_ADSCs, possibly via secretion of α‐LA and LTF.

## 1 INTRODUCTION

Liver fibrosis is the end stage of most of liver diseases and is a serious health care problem worldwide.^1^ In the damaged liver, hepatic stellate cells (HSCs) are activated and produce excessive extracellular matrix components (ECM).^2^ Transforming growth factor (TGF)‐β is a key mediator of hepatic fibrosis and triggers the activation of HSCs, induces ECM synthesis and increases apoptosis of hepatocytes.^3^

Liver transplantation is still considered to be the most effective treatment for end‐stage liver disease, but has several limitations. Recent studies suggest that adipose derived stem cells (ADSCs) show beneficial effects to improve liver fibrosis.^4^ In addition, fibroblast growth factor (FGF) 21 improves lipid profiles and hepatic steatosis.^5^ To enhance the therapeutic effects of ADSCs on liver fibrosis, we established FGF21‐secreting ADSCs (FGF21_ADSCs) and investigated their effects on thioacetamide (TAA)‐induced liver fibrosis in mice.

## 2 MATERIALS AND METHODS

### 2.1 Animal experiments

Eight‐week‐old C57BL/6 male mice were obtained from the Orient Bio Inc (Seongnam, Gyeonggi, Korea). TAA (200 mg/kg; Sigma, St. Louis, MO, USA) was intraperitoneally injected three times a week for 8 weeks. Mice were intravenously injected with either saline, Empty_ADSCs or FGF21_ADSCs (1.5 × 10^6^ cells/mouse). All animal experiments were approved by the Institutional Animal Care and Use Committee at Lee Gil Ya Cancer and Diabetes Institute (IACUC No. LCDI‐2015‐005, LCDI‐2017‐0107). At the end of the experiment, serum alanine transaminase (ALT)/aspartate transaminase (AST) and hyaluronic acid (HA) levels were analysed by an ASAN GOT/GPT test kit (Asan Pharmaceuticals, Hwasung, Gyeonggi, Korea) and ELISA (Corgenix, CO, Westminster, USA), respectively.

### 2.2 Cell culture

LX‐2 cells (Millipore, Temecula, CA, USA) were cultured in high glucose Dulbecco's Modified Eagle's Medium (DMEM) with 10% fetal bovine serum (FBS) and activated with 10 ng/mL TGF‐β1 (100‐21, PEPROTECH, Rocky Hill, NJ, USA) for 24 hours.

### 2.3 Preparation of conditioned medium (CM)

Control or Igk_FGF21 plasmids were transfected into 5 × 10^5^ ADSCs (R7788‐110; Invitrogen, Carlsbad, CA, USA). After 1 day, the medium was replaced with 1% FBS‐DMEM. After 24 hours, medium was filtered and mixed with an equal volume of 1% FBS‐DMEM.

### 2.4 Histological analysis

Hematoxylin and eosin (H&E) or Masson's trichrome staining was carried out using paraffin‐embedded sections. For immunofluorescence staining, liver sections were blocked and incubated with primary antibodies and fluorescence‐conjugated secondary antibodies. DAPI was used for nuclear counterstain. Antibodies used for staining are listed in Table S1.

### 2.5 Western blot analysis

Total proteins were extracted, separated by 8%‐12% SDS‐PAGE and transferred to membranes. After blocking, membranes were incubated with primary antibodies and horseradish peroxidase‐conjugated secondary antibodies. Signals were detected and quantified with ChemiDoc XRS+ system using Image Lab software (Bio‐Rad, Hercules, CA, USA) with the Immobilon Western Chemiluminescent HRP Substrate (Millipore). Antibodies used for western blotting are listed in Table S1.

### 2.6 Secretome analysis

Proteins were digested and analysed by Q‐Exactive orbitrap hybrid mass spectrometer (Thermo Fisher Scientific, San Jose, CA, USA) with Easy‐Nlc 1000 system as described previously.^6^

### 2.7 Statistical analysis

All data are expressed as mean ± SEM of at least three independent experiments. All statistics were performed using one‐way ANOVA (SPSS 19.0 statistical software, IBM, Armonk, NY, USA). *P*‐values less than 0.05 were considered statistically significant.

## 3 RESULTS

### 3.1 Effect of FGF21_ADSCs transplantation on amelioration of liver fibrosis

**Figure 1:**
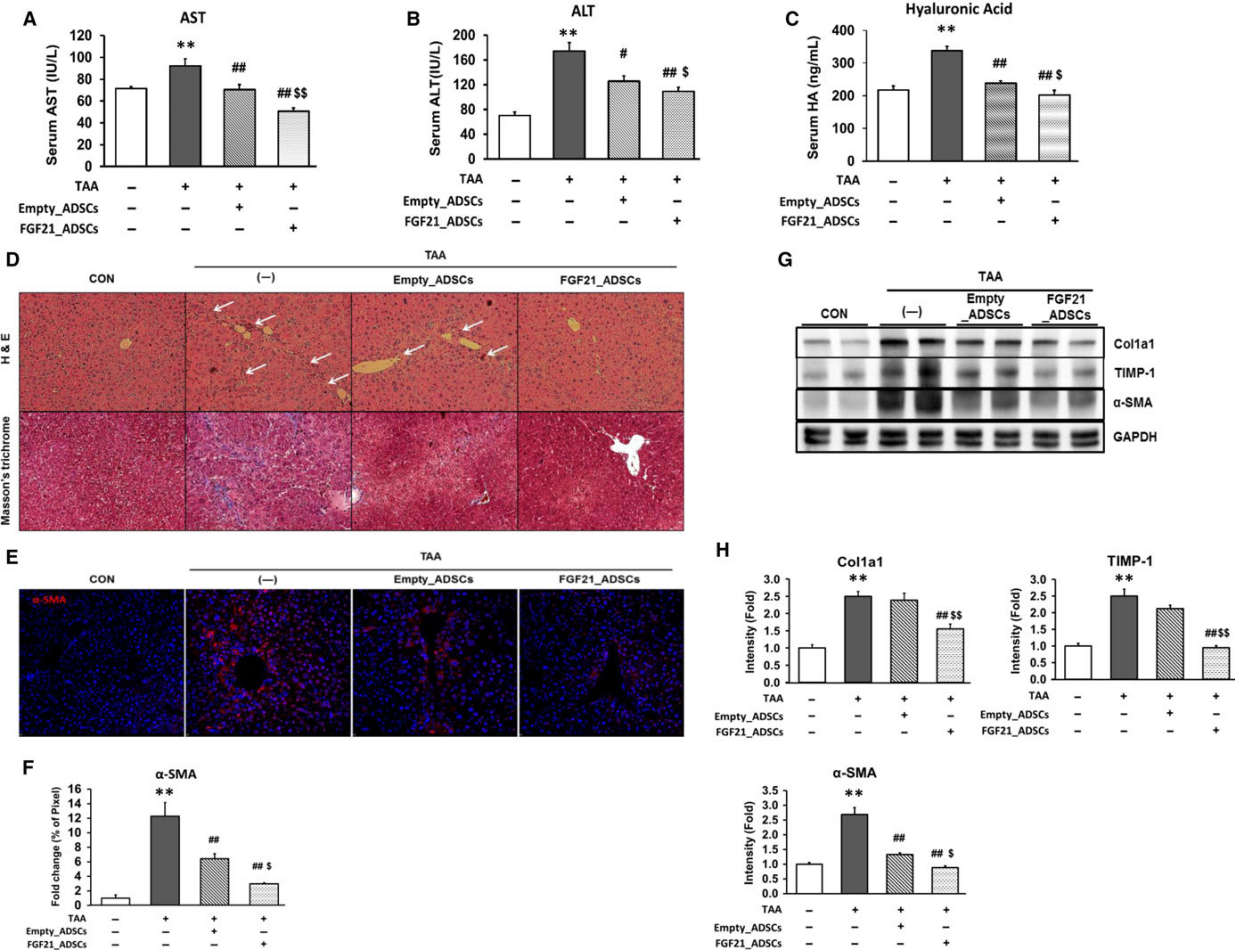
Effect of FGF21_ADSCs transplantation on hepatic fibrogenesis in TAA‐induced liver fibrosis in mice. Mice were injected with vehicle or TAA (200 mg/kg/d) three times a week for a total of 8 wk. TAA‐treated mice were transplanted with vehicle (—), Empty_ADSCs or FGF21_ADSCs by the tail vein. Levels of serum (A) ALT, (B) AST and (C) Hyaluronic acid were measured at 4 wk after transplantation. D, Histological staining of liver sections was carried out. Representative H&E stained and Masson's trichrome stained images are shown. White arrows indicate fibrotic areas (Magnification; 200×). E, Immunofluorescence staining and (F) quantification of α‐SMA (n = 3/group). The percentages of areas for the immunostaining of total image were measured by imageJ and expressed as relative values to CON liver. G, Representative western blot and (H) densitometric analysis for α‐SMA, TIMP‐1 and Col1a1 (n = 6‐13/group). GAPDH was used as control for normalization of results. Data are means ± SEM. **P < 0.01, *P < 0.05. vs untreated mice (CON); ^##^ *P* < 0.01, ^#^ *P* < 0.05. vs TAA (—); ^$$^ *P* < 0.01, ^$^ *P* < 0.05 vs TAA+ Empty_ADSCs

We constructed the Igκ_FGF21 plasmid to facilitate the secretion of FGF21 and established FGF21‐secreting ADSCs (FGF21_ADSCs, Figure S1A‐D). Intravenously transplanted cells reached the liver as confirmed by the expression of FGF21 in the livers from FGF21_ADSC‐injected mice (Figure S1E). TAA was intraperitoneally injected into C57BL/6 mice at 200 mg/kg three times a week for 8 weeks to induce hepatic fibrosis. TAA injection significantly increased ALT and AST, liver damage markers and HA levels, a clinical index of fibrosis, compared with the vehicle‐injected group (Figure S2). Transplantation of Empty_ADSCs (1.5 × 10^6^ cells/mouse) into the tail vein of TAA‐induced fibrosis mice significantly reduced serum ALT, AST and HA levels, and FGF21_ADSCs transplantation showed a further reduction (Figure 1A‐C). The fibrotic area and collagen deposition were significantly increased in liver tissue of the TAA‐induced fibrosis group, reduced by transplantation of Empty_ADSCs and further reduced by FGF21_ADSCs transplantation (Figure 1D). Both Empty_ADSCs and FGF21_ADSCs transplantation improved liver fibrosis, and FGF21_ADSCs transplantation was more effective.

We examined the expression of α‐SMA, which is correlated with the development of fibrosis. Immunofluorescent staining showed that the number of α‐SMA‐stained cells in the TAA‐induced fibrosis group was increased. This increase was reduced by Empty_ADSCs transplantation and further reduced by FGF21_ADSCs transplantation (Figure 1E,F). TIMP‐1 and Collagen type I alpha 1 (Col1a1) protein expression was increased by TAA treatment, and this increase was significantly reduced by FGF21_ADSCs transplantation, but not by Empty_ADSCs transplantation. The α‐SMA protein expression was reduced by Empty‐ADSCs transplantation and further reduced in the FGF21_ADSC‐transplanted group (Figure 1G,H).

### 3.2 Effect of FGF21_ADSCs conditioned media on TGF‐β1‐induced activation of HSCs

**Figure 2:**
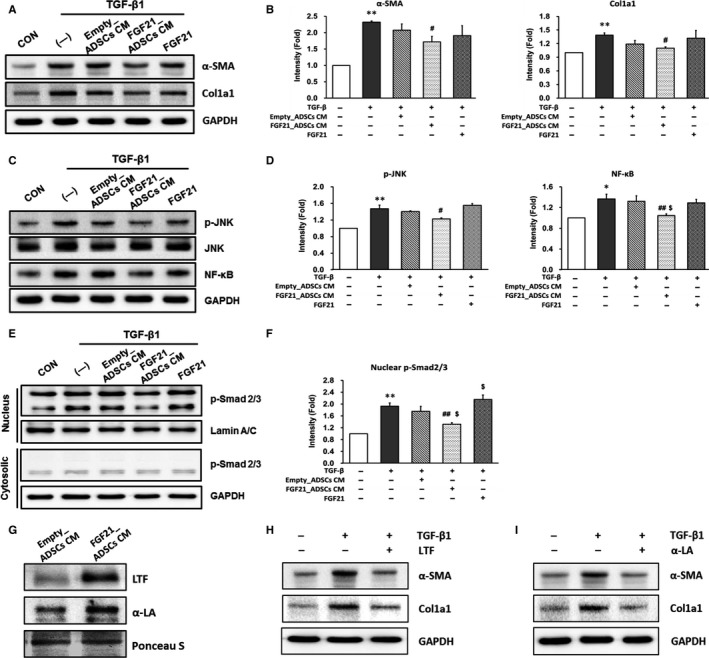
Effect of FGF21_ADSCs CM on the expression of fibrosis markers in LX‐2 cells and secretome analysis of FGF21_ADSCs culture media. LX‐2 cells were untreated (—) or treated with FGF21_ADSCs CM, Empty_ADSCs CM or 200 pg/mL of FGF21 for 24 h in the presence of TGF‐β1. (A, C, E) Representative western blot and (B, D, F) densitometric analysis for (A, B) α‐SMA and Col1a1, (C, D) p‐JNK, JNK and NF‐κB, (E, F) p‐Smad2/3 and Smad2/3 (n = 3/group). GAPDH or Lamin A/C was used as control for normalization of results. Data are means ± SEM. **P < 0.01, *P < 0.05. vs untreated cells (CON); ^##^ *P* < 0.01, ^#^ *P* < 0.05. vs TGF‐β1 (—); ^$$^ *P* < 0.01, ^$^ *P* < 0.05 vs TGF‐β1 with Empty_ADSCs CM. G, Representative western blot of LTF and α‐LA in Empty_ADSCs CM and FGF21_ADSCs CM. Representative western blot of α‐SMA and Col1a1 (n = 3/group) in TGF‐β treated LX‐2 cells incubated with 100 μg/mL of (H) LTF or (I) α‐LA

We determined whether factors secreted from FGF21_ADSCs affect HSC activation in LX‐2 cells using CM from Empty_ADSCs or FGF21_ADSCs during activation with recombinant TGF‐β1. Treatment with FGF21_ADSCs CM, but not Empty_ADSCs CM, significantly inhibited TGF‐β1‐induced expression of α‐SMA and Col1a1 proteins. α‐SMA and Col1a1 protein expression was not different between TGFβ1‐treated cells incubated with or without recombinant FGF21 (200 pg/mL FGF21, the average concentration secreted in the media from FGF21_ADSCs CM) (Figure 2A,B). These results suggest that soluble factors from FGF21_ADSCs, other than FGF21, might contribute to the inhibitory effect on HSC activation.

### 3.3 Regulation of JNK and Smad2/3 signalling by FGF21_ADSCs

JNK and NF‐κB signalling pathways are involved in the expression of α‐SMA and collagen.^7^, ^8^ Protein levels of p‐JNK and NF‐κB were increased in LX‐2 cells activated by TGF‐β1, and this increase was inhibited by FGF21_ADSCs CM treatment but not by Empty_ADSCs CM or 200 pg/mL FGF21 (Figure 2C,D). Similarly, p‐JNK and NF‐κB protein levels were increased in the liver tissues of the TAA‐induced fibrotic group, and these levels were decreased by Empty_ADSCs transplantation and further decreased by FGF21_ADSCs transplantation (Figure S3A,B).

Phosphorylated Smad2/3 (p‐Smad2/3), another key molecule in the TGF‐β signalling pathway,^9^ was increased significantly in TGF‐β‐activated LX‐2 cells. Treatment with FGF21_ADSCs CM significantly inhibited this increase, but there were no changes by treatment with Empty_ADSCs CM or FGF21 (Figure 2E,F). Similarly, we found that p‐Smad2/3 levels in liver tissue were increased in the TAA‐induced fibrotic group, and FGF21_ADSCs transplantation, but not Empty_ADCSs transplantation, significantly inhibited TAA‐induced p‐Smad2/3 (Figure S3C,D).

### 3.4 Secretome analysis of FGF21_ADSCs CM

Secretome analysis by mass spectrometry identified 365 proteins in FGF21_ADSCs CM, of which 33 showed a significant increase and 39 showed a significant decrease as compared with Empty_ADSCs CM (data not shown). Among the proteins that were increased over 2‐fold (Figure S4), we chose lactotransferrin (LTF) and α‐lactoalbumin (α‐LA) for further analysis, because they inhibit liver fibrosis.^10^, ^11^ Western blot analysis showed that LTF and α‐LA secretion was significantly increased in FGF21_ADSCs CM compared with Empty_ADSCs CM (Figure 2G). When we treated LX‐2 cells with LTF or α‐LA in the presence of TGF‐β1, the TGF‐β1‐induced expression of α‐SMA and Col1a1 was inhibited (Figure 2H,I). These results indicate that secretory factors such as α‐LA and LTF, which are altered in FGF21_ADSCs, can improve hepatic fibrosis compared to Empty_ADSCs.

## 4 DISCUSSION

FGF21_ADSCs transplantation by intravenous injection into mice with TAA‐induced liver fibrosis resulted in the recovery of impaired liver function, and this therapeutic effect was greater than Empty_ADSCs. TGF‐β activates HSCs and promotes liver fibrosis progression.^12^ Collagen, TIMP‐1 and α‐SMA are TGF‐β target genes, and are expressed via the JNK and Smad2/3 signalling pathway.^13^ Our results showed that ADSCs transplantation reduced TAA‐induced JNK and Smad2/3 activation in vivo, with more potent inhibition by transplantation of FGF21_ADSCs than Empty_ADSCs.

The therapeutic effect of MSC therapy is due to a variety of biologically active factors produced by them.^14^ Our study showed that CM from FGF21_ADSCs significantly inhibited TGF‐β‐induced expression of fibrogenic factors compared with CM from Empty_ADSCs. Interestingly, when LX‐2 cells, a human HSC line, were incubated with the same amount of FGF21 as found in the CM of FGF21_ADSCs, there was almost no effect on the inhibition of JNK and Smas2/3 activation, suggesting that soluble factors other than FGF21 might contribute to this stronger inhibitory effect. Among the secreted proteins upregulated in the FGF21_ADSCs CM, we noted that α‐LA and LTF might be involved in the inhibition of fibrogenic activity. α‐LA and LTF have a liver protective and anti‐fibrotic effect, respectively.^10^, ^11^ We confirmed that α‐LA or LTF treatment inhibited TGF‐β‐induced α‐SMA and Col1a1 expression in LX‐2 cells. Therefore, α‐LA and LTF secreted by FGF21_ADSCs might contribute in part to the potent inhibitory effect of these cells on liver fibrosis in our model.

## CONFLICT OF INTEREST

The authors confirm that there are no conflicts of interest.
