# Supplementary figures and images for: Effects of FGF21‐secreting adipose‐derived stem cells in thioacetamide‐induced hepatic fibrosis
Source: J Cell Mol Med. 2018 Jul 18;22(10):5165–9. doi: 10.1111/jcmm.13795 (PMC6156392; doi:10.1111/jcmm.13795)

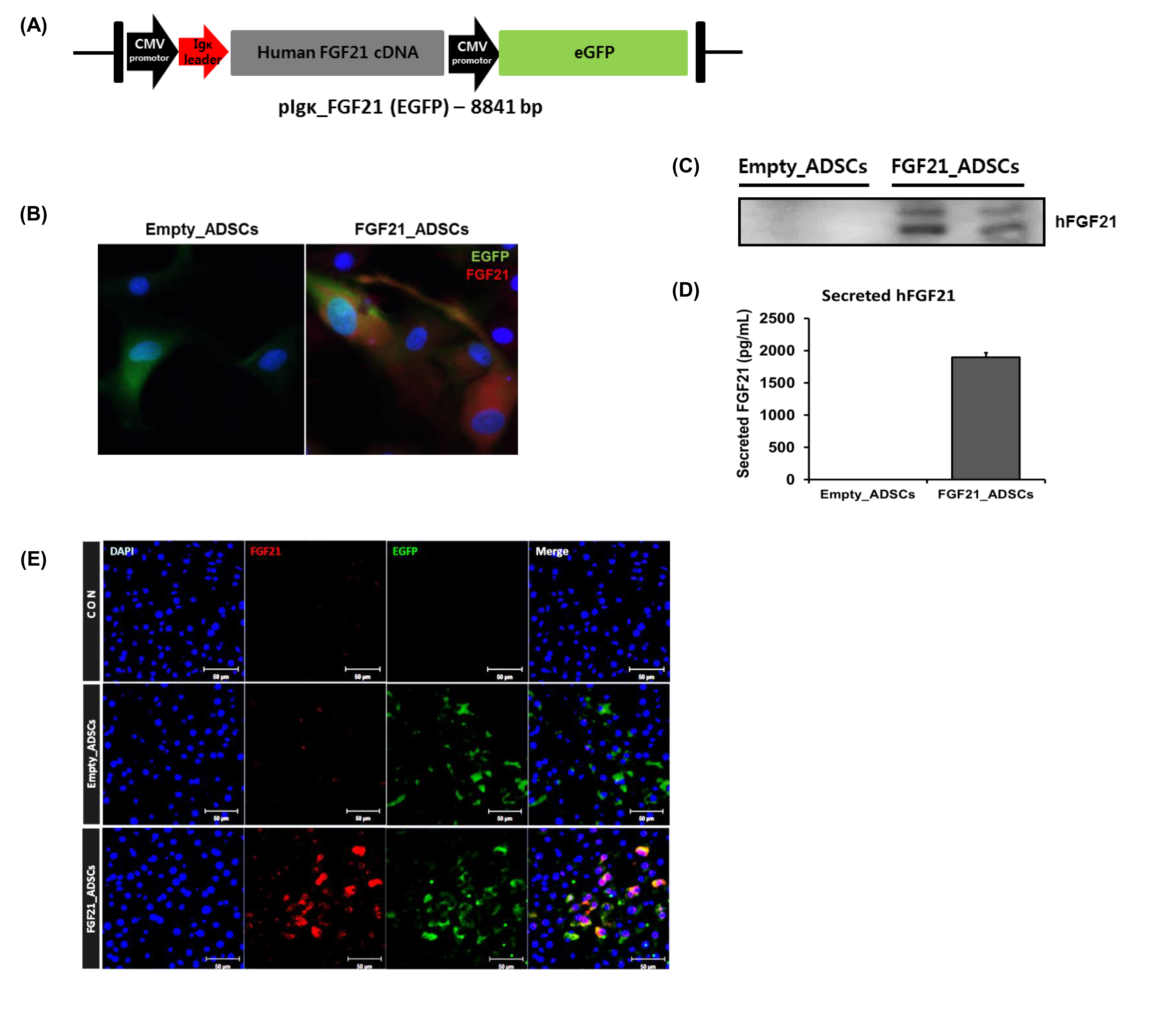

Supplement: Supplementary file 1 [file JCMM-22-5165-s001.tif]

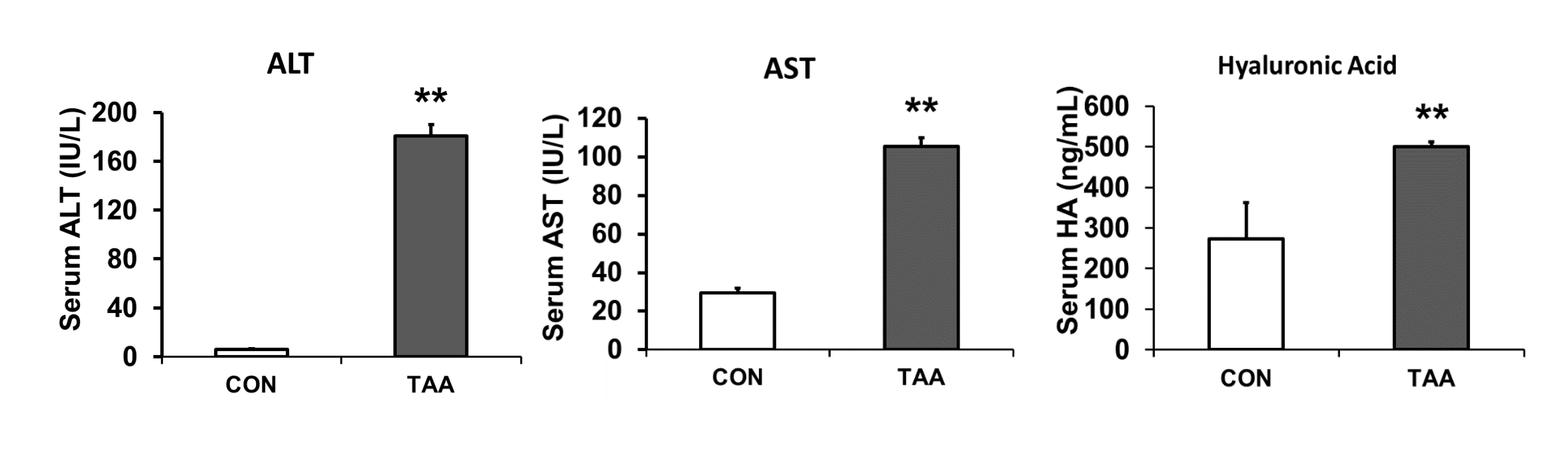

Supplement: Supplementary file 2 [file JCMM-22-5165-s002.tif]

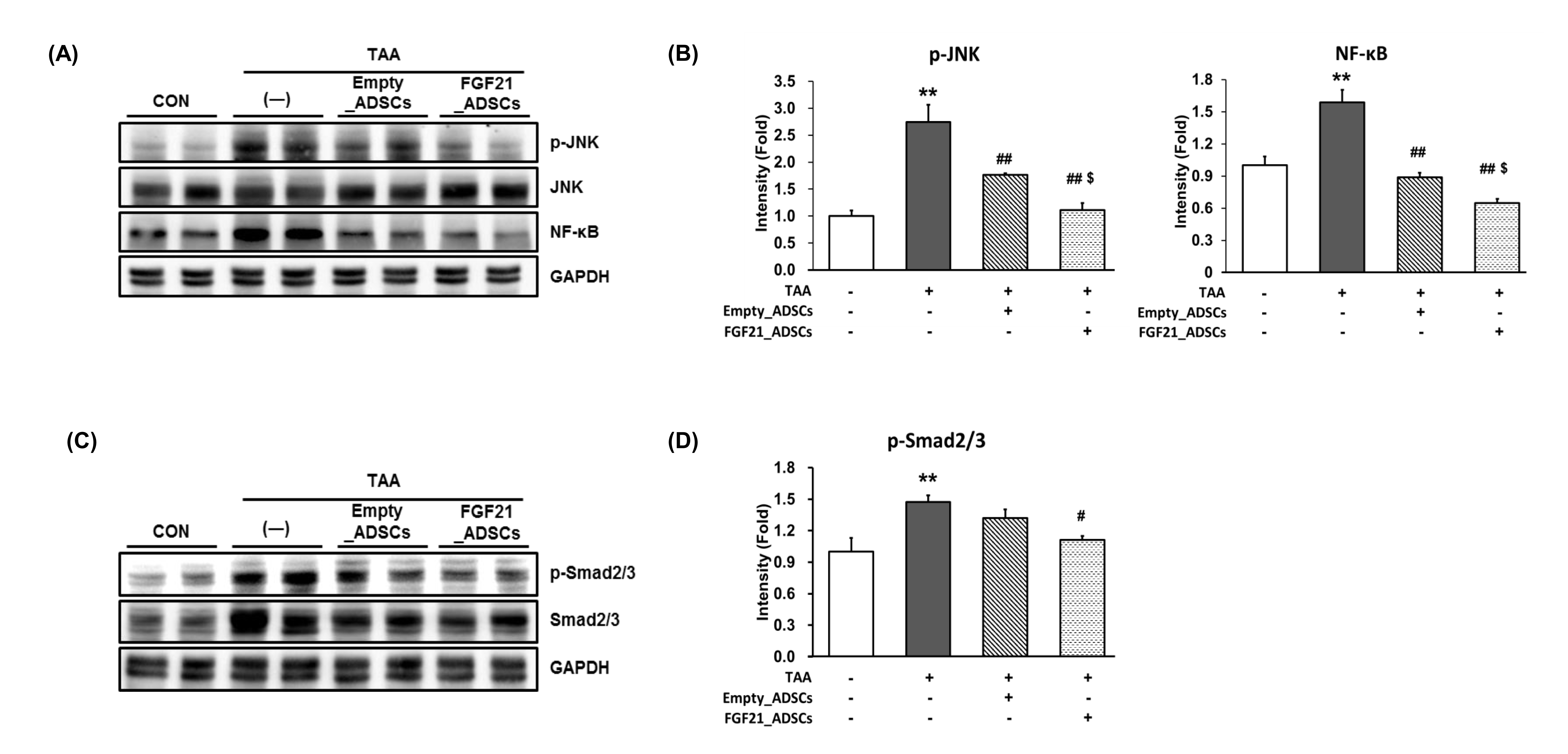

Supplement: Supplementary file 3 [file JCMM-22-5165-s003.tif]

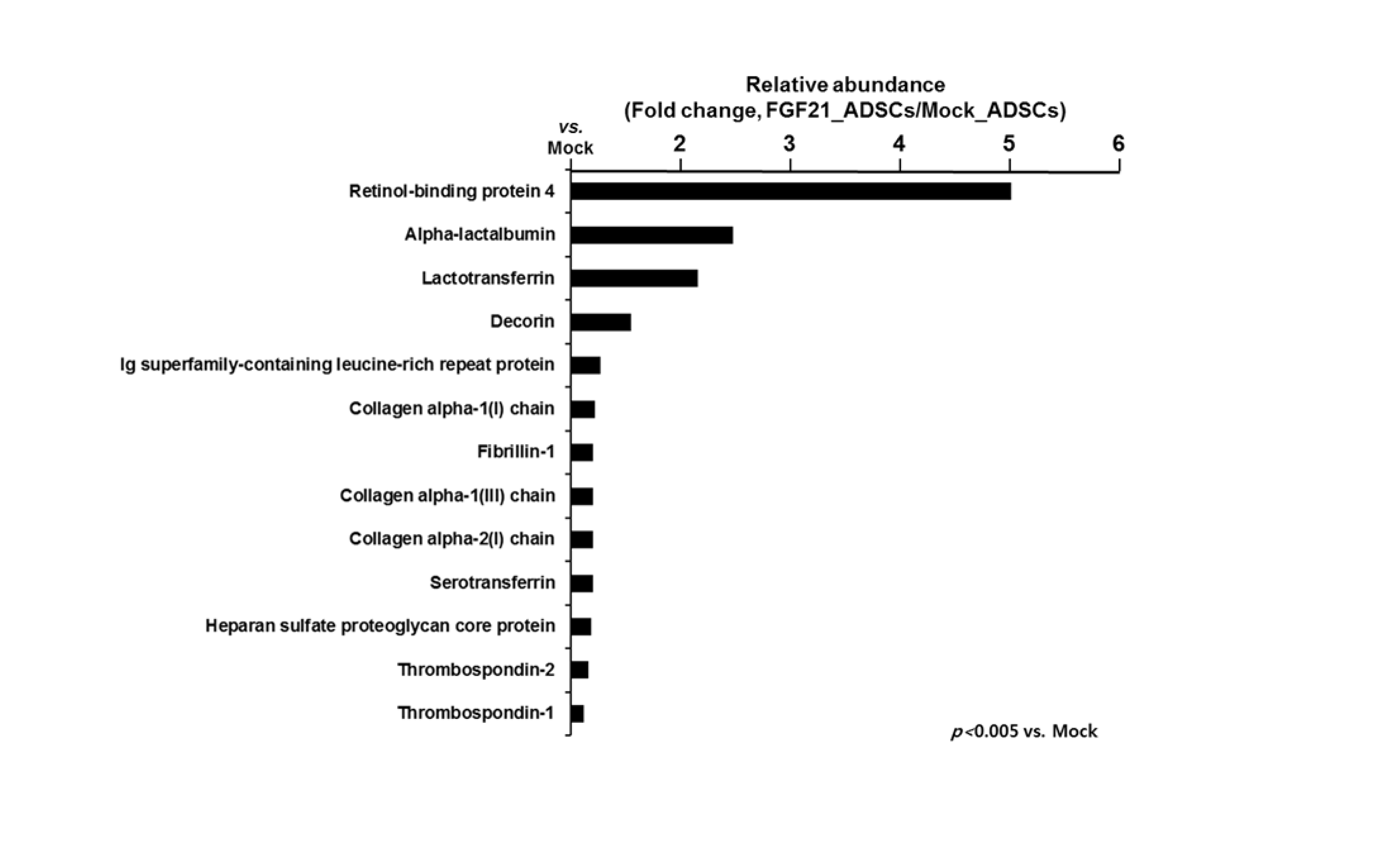

Supplement: Supplementary file 4 [file JCMM-22-5165-s004.tif]
