# Supplementary material for: Effects of FGF21‐secreting adipose‐derived stem cells in thioacetamide‐induced hepatic fibrosis
Source: J Cell Mol Med. 2018 Jul 18;22(10):5165–9. doi: 10.1111/jcmm.13795 (PMC6156392; doi:10.1111/jcmm.13795)
Supplement: Supplementary file 5 [file JCMM-22-5165-s005.docx]

**Materials and Methods**

*Establishment of FGF21-secreting ADSCs*

Human FGF21 cDNA was synthesized (NM_019113.3; COSMO GeneTech, Seoul, Korea) and inserted into the pSecTag2B vector (INV-V900-20, Invitrogen, CA, USA). FGF21 expressed from this vector was fused to the Igκ signal peptide to facilitate secretion. To verify transfection and tracking of transfected cells, the enhanced green fluorescent protein gene (6083-1, Addgene, MA, USA) was cloned into the AAVS1 donor vector (TGAAVS1, ToolGen, Seoul, Korea)*.* Human ADSCs (R7788-110; Invitrogen) were cultured in MesenPRO RS™ Medium (E07-1000, Invitrogen) in 5% CO_2_ at 37 °C, and 5×10^5^ ADSCs were transfected with 5 μg of Igκ_FGF21 (FGF21_ADSCs) or vector without the Igκ_FGF21 cDNA fragment (Empty_ADSCs) by electroporation (at 1005 V, 35 ms, 2 pulses) using the Neon^®^ Transfection System (MPK10096, Invitrogen) according to the manufacturer's guidelines and incubated for 2 days. The expression and secretion of FGF21 were confirmed by western blot and FGF21 ELISA (DF2100, RnD systems, MN, USA) respectively. Empty_ADSCs were used as a control.

**Supplementary Figure legends**

**Supplementary Figure. 1. Establishment of FGF21-secreting ADSCs (FGF21_ADSCs).** (A) Schematic diagram of the pIgκ_FGF21 construct. ADSCs were transfected with pIgκ_FGF21 (FDF21_ADSCs) or a control plasmid without Igκ_FGF21 DNA (Empty_ADSCs). At 48 h after transfection, (B) immunofluorescence staining was performed with anti-FGF21 antibody (red) and GFP (green); Magnification 400×. (C) Western blot analysis, and (D) ELSIA for secreted FGF21 in culture media were performed. (E) Mice were injected with FGF21_ADSCs or Empty_ADSCs and 3 days later, livers were obtained for immunohistochemical staining. Scale bar, 50 μm.

**Supplementary Figure 2. Identification of TAA-induced fibrosis through ALT, AST and HA levels in serum.** Mice were injected with vehicle (CON) or TAA (200 mg/kg/day) three times a week for a total of 8 weeks. Serum ALT, AST and hyaluronic acid levels were measured for identification of TAA-induced fibrosis. Data are means ± SEM. ***p*<0.01 *vs.* CON.

**Supplementary Figure 3. Effect of FGF21_ADSCs on JNK and Smad signaling pathway in TAA-induced liver fibrosis in mice.** Mice were injected with vehicle or TAA (200 mg/kg /day) three times a week for a total of 8 weeks. TAA-treated mice were transplanted with vehicle (―), Empty_ADSCs or FGF21_ADSCs by the tail vein. At 4 weeks after cell transplantation, liver tissues were collected. (A, C) Representative western blot and (B, D) densitometric analysis of (A, B) p-JNK, JNK and NF-κB, (C, D) p-Smad2/3 and Smad2/3 (n=3/group). GAPDH was used as control for normalization of results. Data are means ± SEM. ***p*<0.01, **p*<0.05. *vs.* untreated cells (CON); ^##^*p*<0.01, ^#^*p*<0.05. *vs.* TAA (―); ^$^*p*<0.05 *vs.* TAA+Empty_ADSCs.

**Supplementary Figure 4. Secretome analysis of FGF21_ADSCs culture media.** Secretome analysis was performed on FGF21_ADSCs CM and Empty_ADSCs CM by mass spectroscopy. Factors that showed a significant (*p*<0.005) increase in the FGF21_ADSCs CM compared with the Empty_ADSCs CM are listed.

*Supplementary table 1. List of antibodies*

| Antigen | Manufacture | Cat. No. |
| --- | --- | --- |
| α-SMA | Abcam | Ab7817 |
| GFP | Invitrogen | a-11122 |
| FGF21 | SantaCruz | Sc-16842 |
| TGF-β1 | Cell signaling | 3771 |
| JNK | Cell signaling | 9252 |
| p-JNK | Cell signaling | 9251 |
| Smad2/3 | Cell signaling | 5678 |
| p-Smad2/3 | Cell signaling | 8828 |
| α -LA | Bethyl Laboratories | A10-128A |
| LTF | Bethyl Laboratories | A80-143A |
| TIMP-1 | SantaCruz | Sc-21734 |
| NF-κB | SantaCruz | Sc-372 |
| Col1a1 | SantaCruz | sc-293182 |
| Lamin A/C | Cell signaling | 4777 |
| GAPDH | Millipore | MAB374 |
